# Depression score and body mass index mediate the association between dietary vitamin C intake and female infertility: a study based on NHANES 2013–2018

**DOI:** 10.3389/fnut.2025.1650311

**Published:** 2025-09-10

**Authors:** Lingxin Zheng, Qingling Ren, Yong Tan

**Affiliations:** The First Clinical Medical College, Nanjing University of Chinese Medicine, Nanjing, Jiangsu, China

**Keywords:** dietary vitamin C intake, female infertility, NHANES database, cross-sectional studies, depression score, body mass index

## Abstract

**Background:**

The supplement of antioxidants to improve fertility has received widespread attention. The efficacy of the dietary antioxidant vitamin C (VC) on female infertility has not been fully elucidated. This study investigates the relationship between VC intake and infertility in terms of depression score and body mass index (BMI).

**Methods:**

Our cross-sectional study included 2,381 adult American females aged from 18 to 44 from the National Health and Nutrition Examination Survey (NHANES, 2013–2018) database. Multiple logistic regressions, smoothed curve fitting, mediation and sensitivity analysis were conducted to describe the relationship between VC intake and infertility.

**Results:**

Compared to the low VC intake group (Q1), the probability of female infertility in the highest group (Q4) decreased by 39.4% (adjusted OR = 0.606; 95% CI: 0.419, 0.878, *P* = 0.008). A threshold non-linear association between VC and infertility was more significant in women aged from 18 to 34 (*P* = 0.033). Moreover, the relationship between VC and infertility was mediated by 5.28% depression (*P* = 0.034) and 7.83% BMI (*P* = 0.010), respectively.

**Conclusions:**

The protective effect of VC on female fertility was most significant in the group with the highest VC intake. The association between VC intake and the likelihood of female infertility was non-linear and smallest infertility index occurred when VC intake reached 132.7 mg in women aged 18–34. Depression score and BMI seemed to mediate the relationship. However, further research was needed to confirm the interaction effects of VC, depression and BMI on female infertility from basic and clinical perspectives.

## Introduction

Infertility is a disease defined as the inability to have a clinical pregnancy after 12 months of regular and unprotected sexual intercourse (1, 2). According to the latest report released by the World Health Organization (WHO), approximately 17.5% of adults worldwide are affected by infertility. In the United States, the proportion of reproductive aged women who seek infertility treatment accounts for approximately 12.7% each year (3). Female infertility is a public health issue of worldwide concern affecting millions of individuals and couples worldwide. Despite its prevalence, the determinants of infertility have not been fully elucidated, involving a combination of biological, psychological, lifestyle, and socio-economic factors (4–8).

Dietary and nutritional factors play an essential role in maintaining normal reproductive function through epigenetic mechanisms to imprint the human genome (5, 9). Recently, mounting evidence attests that dietary nutrition status might be more crucial for the infertile to ameliorate pregnancy outcomes (10). As one of the common dietary nutrients, VC is an efficient antioxidant used to alleviate oxidative stress caused by ascorbic acid peroxidase. Showell et al. discovered that the utilization of antioxidants might increase the clinical pregnancy rate (11). In 1989, the Developmental Origins of Health and Disease (DOHaD) theory revealed that nutrition supplements in childhood were possibly related to the risk of chronic diseases in adulthood. DiTroia et al. manifested that VC supplements during pregnancy are vital for the development of female fetal germ cells (12). In a high-quality clinical study, increased VC intake was associated with a shorter time to pregnancy, but its effectiveness relied on BMI and age (13).

With the improvement of living standards and the increase of work pressure, the phenomenon of obesity and psychological disorders has become more prevalent. Most scholars currently believe that obesity has a negative impact on female reproductive health (14). As the primary index for obesity, BMI was often used in clinical practice to assess the risk of related diseases (15). The latest high-quality research showed that obese individuals might need to consume more VC because oxidative stress (OS) can significantly increase the likelihood of obesity. According to this article, for every 10 kilograms of weight gained, a 10 mg VC intake was additionally required daily within the range of 60–90 kg. Therefore, plasma VC concentrations of the obese can be more similar to thinner individuals (16). Additionally, it had been reported that women with depressive symptoms were more prone to suffer from infertility due to the fluctuations in sexual hormone secretion affected by emotional disorders (17). The Patient Health Questionnaire 9 (PHQ-9) was a reliable method to measure the degree of depression, which was widely recognized in clinical studies (18). Obviously, many infertile women with depressive symptoms affected their clinical pregnancy rate, and terrible pregnancy outcomes were compounded by the psychological burden on patients (19). The latest research has found that the depression-like behavior was based on stress response to abnormal DNA methylation. The imbalance of endogenous VC homeostasis might play an essential role in the occurrence of depression (20).

This cross-sectional study is mainly based on the NHANES database in the United States from 2013 to 2018. Multivariate logistic regression was used to explore the association between female infertility and VC intake. Mediation and sensitivity analysis was conducted on the study to clarify the linkage between VC intake and infertility. Therefore, the purpose of this study is to investigate the relationship between VC intake and infertility in different age groups.

## Materials and methods

### Data sources

**Figure 1:**
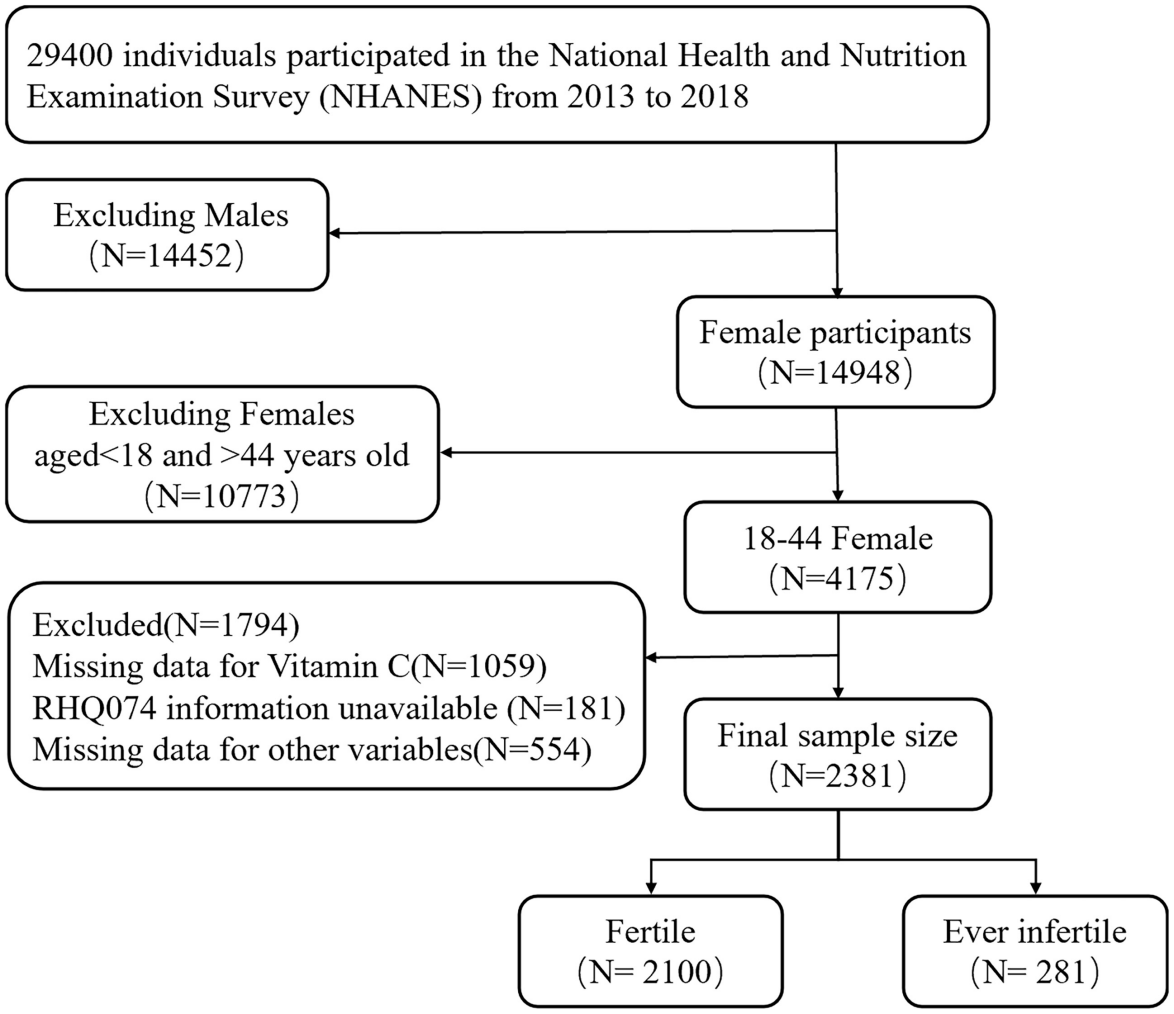
Flow chart of procedures from identification of eligible patients to final inclusion.

The analysis conducted in this study was based on data collected from the NHANES database (https://www.cdc.gov/nchs/nhanes/). As shown in Figure 1, inclusion criteria were as follows: 1. Female participants aged 18–44. 2. Statistical information on NHANES participants' fertility status (variable name: RHQ074), VC intake dietary recall interviews (variable name: DR1TVC, DR2TVC), and other covariates were collected in the NHANES database. Exclusion criteria were as follows: 1. participants lacking the dietary interview data on VC intake was excluded (*N* = 1,059). 2. A few participants were missing information about whether they were ever infertile (*N* = 181). 3. There was some unavailable data for other variables (*N* = 554). Eventually, 2,381 adult American females aged from 18 to 44 was included.

### Exposure and outcomes

The exposure variable of this study was VC. Two 24-h dietary recall interviews were conducted for NHANES participants. Two 24-h dietary recall interviews were conducted for NHANES participants. Intakes of foods in the previous 24 h were collected in person in the MEC (day 1) and by telephone 3–10 d later (day 2). Average VC daily intakes were calculated from 2 days of dietary recall data (variable name: DR1TVC, DR2TVC). The outcome variable of this study was fertility status. All female participants aged 18–44 answered whether they had “Tried for a year to become pregnant” (variable name: RHQ074). According to the different answers, female participants were divided into two groups: fertile and ever infertile.

### Mediator variables and covariates

As shown in directed acyclic graph (Supplementary Figure S1), PHQ-9 score and BMI were mediator variables in this study. Firstly, the PHQ-9 questionnaire consisted of 9 questions with a score of 0–3, respectively. The total score was applied to measure the severity of depressive symptoms, with a threshold of 10 or above indicative of depressive issues. BMI was defined as the value of weight (kg) divided by the height (m) square. It was classified as non-obesity (BMI < 30) and obesity (BMI ≥ 30) based on CDC guidelines. The covariates in this study included age (RIDAGEYR), marital status (DMDMARTL), sleep trouble (SLQ050), diabetes mellitus history, hypertension history, hyperlipidemia history, and physical activity (PAQ620). Comorbidity data (including diabetes, hypertension, and hyperlipidemia) was obtained from questionnaire, examination, and laboratory data. Average daily intakes of vitamin D (VD), vitamin E (VE), folate, magnesium, zinc and selenium intake were estimated based on 2 days of dietary recall data, the same methodology as applied for VC.

### Statistical analysis

Participants were separated into two groups based on whether they had infertility. Firstly, a wilcoxon analysis or chi squared test was used to compare continuous and categorical variable sets, respectively. Secondly, multiple logistic regression models were used to explore the relationship between VC intake and infertility. Smooth curve fitting was performed to explore the non-linearity, and turning points were calculated through threshold effect analysis. Thirdly, subgroup analysis and the test for interaction was included in sensitivity analysis to confirm the stability of the results. And then mediation models of main variables would be established to analyze the total effect (TE), direct effect (DE), and mediation effect (ME). The proportions mediated formula was shown as follows: ME/TE^*^100%. Eventually, except for the independent and dependent variables, we used multiple imputation, based on 5 replications and a chained equation approach method in the R MI procedure, to account for missing data of other remaining variables (*N* = 554). All statistical analyses were done in EmpowerStats 4.2 (http://www.empowerstats.com), Storm Statistical Platform (http://www.medsta.cn/software) and R software version 4.3.1; *P* < 0.05 was considered significant.

## Results

### Baseline characteristics of the study population

A total of 2,381 NHANES participants were ultimately involved in this study. Baseline characteristics of the study population were briefly summarized in Table 1. The distributions of age, marital status, diabetes mellitus history, hypertension history, hyperlipidemia history, sleep trouble, BMI, PHQ-9 score, VC intake significantly differed between fertile and ever infertile participants. To more comprehensively explore healthy nutritional patterns, we initially examined intakes of VD, VE, folate, magnesium, zinc, and selenium. Subsequent analysis focused on VC after no significant associations were found for other nutrients (Supplementary Table S1).

**Table 1:** Descriptions of individuals' characteristics.

**Variable**	**Fertile (*n* = 2,100)**	**Ever infertile (*n* = 281)**	***P*-value**
Age (years) (%)			<0.001
18–34	1,264 (60.19%)	127 (45.20%)	
35–44	836 (39.81%)	154 (54.80%)	
Race/ethnicity (%)			0.189
Mexican American	348 (16.57%)	46 (16.37%)	
Non-Hispanic White	714 (34.00%)	110 (39.15%)	
Non-Hispanic Black	458 (21.81%)	63(22.42%)	
Other	580 (27.62%)	62 (22.06%)	
Married/live with partner (%)			<0.001
Yes	1,191 (56.71%)	205 (72.95%)	
No	909 (43.29%)	76 (27.05%)	
Education level (%)			0.725
Below high school	308 (14.67%)	39 (13.88%)	
High school or above	1,792 (85.33%)	242 (86.12%)	
Poverty income ratio (%)			0.114
Poor	532 (25.33%)	59 (21.00%)	
Not poor	1,568 (74.67%)	222 (79.00%)	
Diabetes mellitus history (%)			0.009
Yes	113 (5.38%)	26 (9.25%)	
No	1,987 (94.62%)	255 (90.75%)	
Hypertension history (%)			0.008
Yes	281 (13.38%)	54 (19.22%)	
No	1,819 (86.62%)	227 (80.78%)	
Hyperlipidemia history (%)			0.008
Yes	256 (12.19%)	50 (17.79%)	
No	1,844 (87.81%)	231 (82.21%)	
BMI	29.59 ± 8.31	32.54 ± 9.37	<0.001
Sleep trouble (%)			0.003
Yes	479 (22.81%)	87 (30.96%)	
No	1,621 (77.19%)	194 (69.04%)	
Physical activity			0.884
Yes	869 (41.38%)	115 (40.93%)	
No	1,231 (58.62%)	166 (59.07%)	
PHQ-9, median (Q1, Q3)	2.00 (0.00–5.00)	3.00 (1.00–6.00)	<0.001
Depression			0.002
Yes	202 (9.62%)	44 (15.66%)	
No	1,898 (90.38%)	237 (84.34%)	
VC (mg), median (Q1, Q3)	59.08 (27.60–105.35)	43.45 (22.90–86.85)	0.003

### Associations between dietary VC intake and infertility

**Figure 2:**
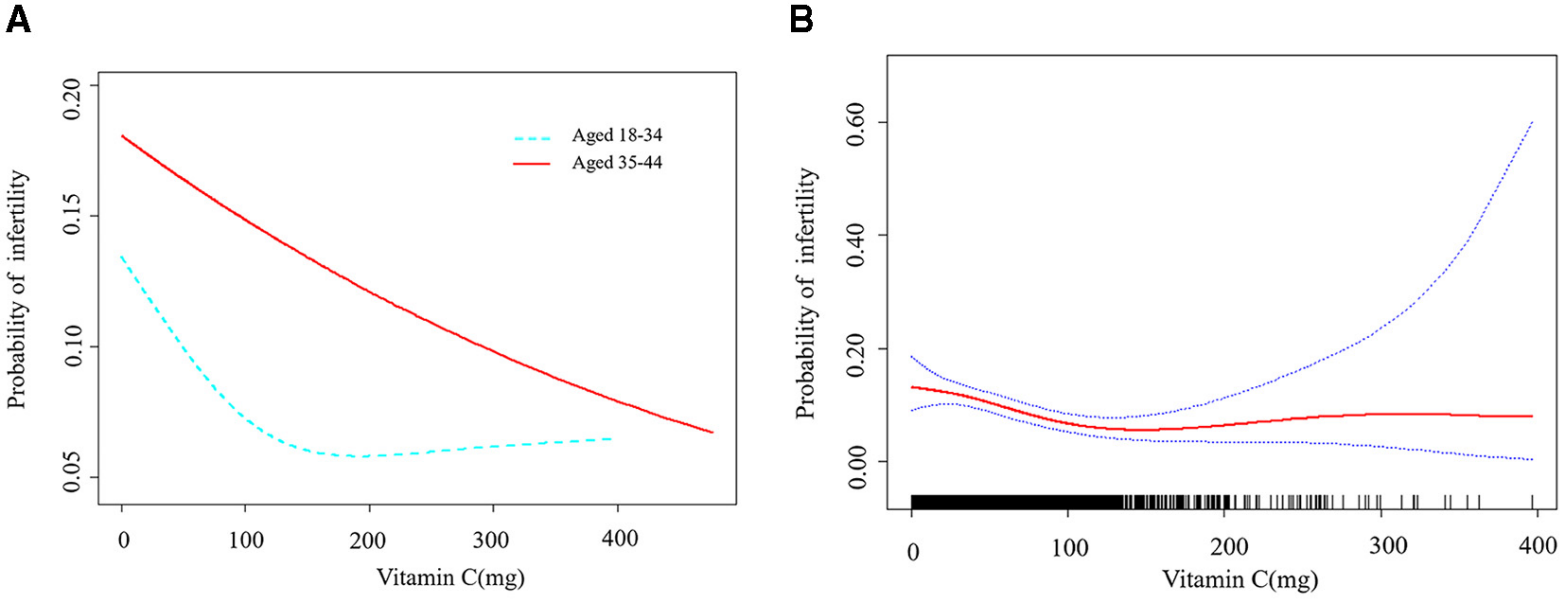
A threshold non-linear association between VC (mg) and infertility. All were adjusted for Marital status; Sleep trouble; Diabetes mellitus history; Hypertension history; Hyperlipidemia history. **(A)** Association between different age groups was found in a generalized additive model (GAM). **(B)** Association in group aged 18 and 34 was found (*P* = 0.022) in a GAM. A solid red line represents the smooth curve fit between variables. Blue bands represent the 95% confidence interval from the fit.

As seen in Table 2, we found that the trend revealed that higher VC intake was related to lower risk of infertility. A significantly higher prevalence of infertility in women was observed in the lowest dietary VC quartile than in those with the highest doses (Model 3, OR: 0.606, 95% CI: 0.419–0.878, *P* = 0.008). According to the literature, age is one of the essential factors in the study of female infertility (3). Age not only affects female fertility, but also closely associated with the effectiveness of infertility treatment. Therefore, age stratification in analysis can provide more precise evidence of relationship between VC intake and infertility. We conducted research models on different age groups to explore the relationship further (Figure 2A). There was a threshold effect between VC intake and infertility rates in the group aged 18–34 with a greater desire to have a child. According to the result of log likelihood ratio test in Table 3, it was confirmed that there was a non-linear association between VC intake and the likelihood of infertility in group aged from 18 to 34. Then, we used the VC intake for curve fitting analysis owing to the skewed data distribution (Figure 2B). The inflection point was calculated as 132.7 mg (VC intake). On the left side of the inflection point, each unit increase in VC intake was associated with a 0.9% reduction in the possibility of infertility (*P* = 0.001). On the right side of the inflection point, the effect size was 1.004 (95% CI: 0.996, 1.012), which elaborated a weak correlation (*P* > 0.05).

**Table 2:** Relationship between VC and infertility in different models.

**Exposure**	**Model 1**		**Model 2**		**Model 3**	
	**OR (95% CI)**	***P*** **-value**	**OR (95% CI)**	***P*** **-value**	**OR (95% CI)**	***P*** **-value**
VC(mg) (continuous)	0.997 (0.994–0.999)	0.004	0.996 (0.994–0.999)	0.002	0.997 (0.994–0.999)	0.004
**Quartile of VC**						
Q1	1.00 (Reference)		1.00 (Reference)		1.00 (Reference)	
Q2	0.986 (0.710–1.370)	0.933	0.926 (0.664–1.293)	0.653	0.969 (0.693–1.355)	0.854
Q3	0.718 (0.505–1.019)	0.064	0.681 (0.478–0.971)	0.034	0.717 (0.502–1.026)	0.069
Q4	0.615 (0.427–0.884)	0.009	0.574 (0.398–0.829)	0.003	0.606 (0.419–0.878)	0.008
*P* for trend	0.002		0.001		0.002	

Model 1 adjusted for: None. Model 2 adjusted for: Age; Marital status. Model 3 adjusted for: Age; Marital status; Sleep trouble; Diabetes mellitus history; Hypertension history; Hyperlipidemia history.

**Table 3:** The results of the two-piecewise linear regression model.

**Outcome**	**Adjusted OR (95% CI)**	***P*-value**
Fitting by linear regression model	0.995 (0.992, 0.999)	0.010
**Fitting by two-piecewise linear regression model**		
Inflection point	132.7	
VC (mg) < 132.7	0.991 (0.986, 0.996)	0.001
VC (mg) > 132.7	1.004 (0.996, 1.012)	0.317
Log likelihood ratio test	0.033	

### Mediation analysis of PHQ-9 score and BMI

**Figure 3:**
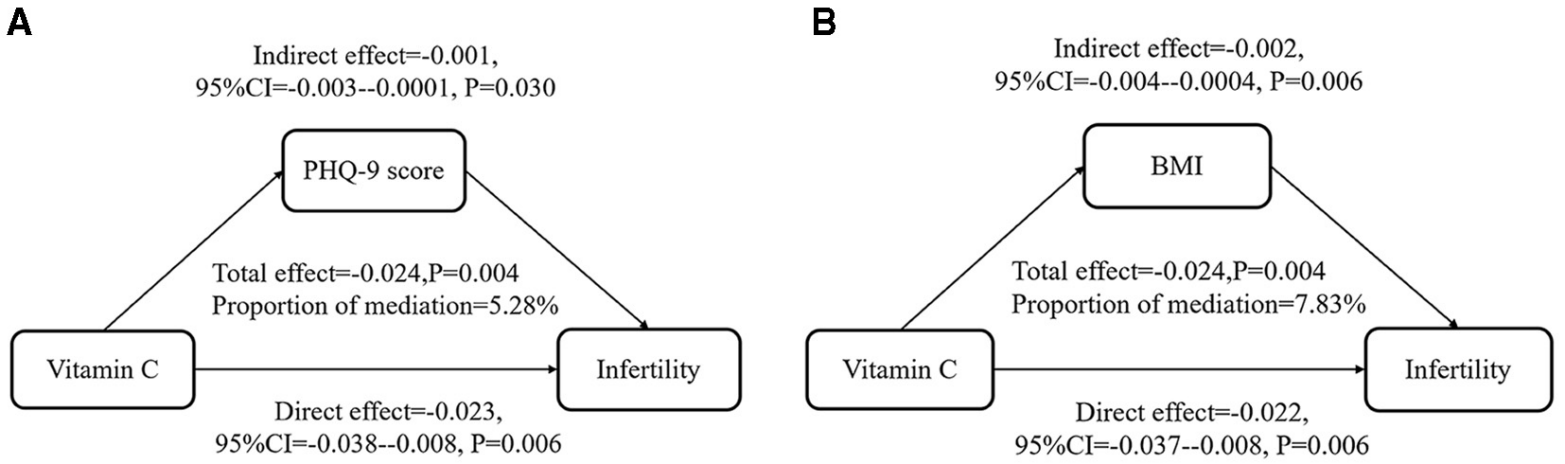
PHQ-9 score and BMI played a mediating role in the relationship between VC intake and infertility. All were adjusted as Model 3. **(A)** PHQ-9 score mediated 5.28% (*P* = 0.034) in the relationship between VC intake and infertility. **(B)** BMI mediated 7.83% (*P* = 0.010) in the relationship between VC intake and infertility.

In the mediation analysis, a total effect estimate of −0.024 was observed (*P* = 0.004), indicating a statistically significant association. The direct effect of VC intake suggested that it had a negative influence on infertility outcomes independent of PHQ-9 score and BMI. In contrast, the mediation effects of PHQ-9 score and BMI were 0.001 and 0.002, respectively, with *P*-values below 0.05, which manifested a modest negative mediation. Notably, dietary VC intake was inversely associated with infertility through PHQ-9 score and BMI in Figure 3. Overall, these results highlighted the significance of VC intake as a protective factor for female fertility, while underlined the limited mediating role of physical and psychological status.

### Sensitivity analysis

**Figure 4:**
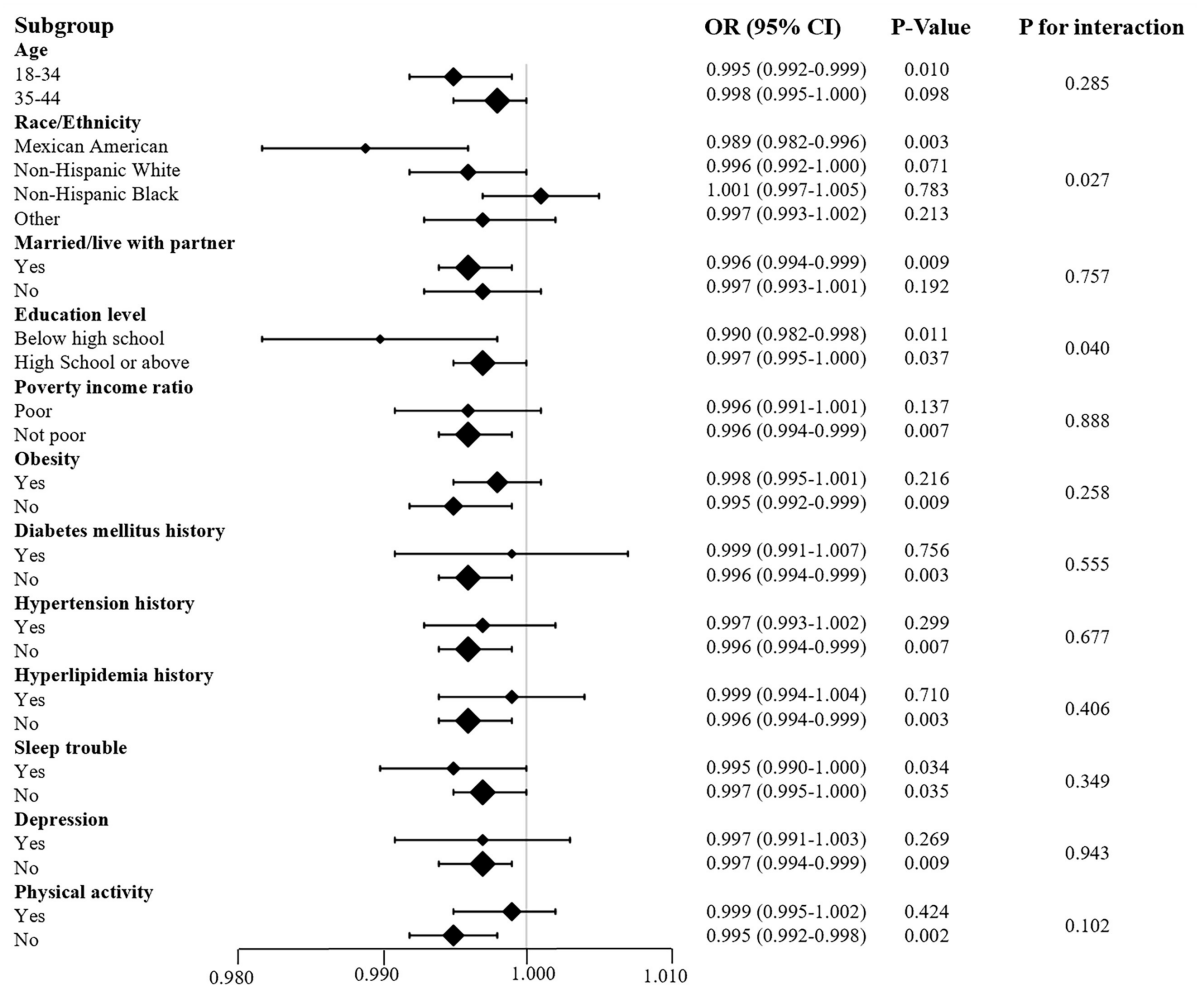
Subgroup analysis of the association between VC and infertility. Each stratification adjusted for all the factors (age; marital status; sleep trouble; diabetes mellitus history; hypertension history; hyperlipidemia history) except the stratification factor itself.

We performed subgroup analysis by individual characteristics, such as age, ethnicity, marital status, education level, and poverty income ratio. Whether they suffered from obesity, diabetes mellitus, hypertension, hyperlipidemia, sleep trouble, depression or lack of physical activity, the participants were also included in the subgroup analysis. This analysis aimed to confirm the stability of the results. The results implied that the relationship between VC and infertility remained stable in these subgroups (Figure 4). The test for interaction indicated that the effects of education level and ethnicity on infertility were significantly influenced by VC intake (P < 0.05). Each unit increase in dietary VC intake influenced the infertility rate inversely by 0.1% approximately in both the Mexican American population and those with education below high school. Moreover, the Alternative analytic strategies yielded consistent results in Table 4, including the cases with missing data, complete cases without missing data, and multiple imputation of missing data.

**Table 4:** Sensitivity analysis of variables with missing data, complete case without missing data and the datasets with imputed variables from multiple imputation.

**Exposure**	**With missing data**		**Complete case**		**Multiple imputation**	
	**OR (95% CI)**	***P*** **-value**	**OR (95% CI)**	***P*** **-value**	**OR (95% CI)**	***P*** **-value**
VC(mg) (continuous)	0.997 (0.994–0.999)	0.002	0.997 (0.994–0.999)	0.004	0.997 (0.995, 0.999)	0.014
**Quartile of VC**						
Q1	1.00 (Reference)		1.00 (Reference)		1.00 (Reference)	
Q2	1.013 (0.730–1.406)	0.937	0.969 (0.693–1.355)	0.854	1.025 (0.743–1.414)	0.880
Q3	0.712 (0.503–1.010)	0.057	0.717 (0.502–1.026)	0.069	0.733 (0.518–1.035)	0.078
Q4	0.638 (0.447–0.911)	0.013	0.606 (0.419–0.878)	0.008	0.648 (0.454–0.927)	0.017

All adjusted for: Age; Marital status; Sleep trouble; Diabetes mellitus history; Hypertension history; Hyperlipidemia history.

## Discussion

Female infertility is a significant global public health issue, intricately linked to living standards and social factors. Recent trends such as increased social and work-related stress, unhealthy lifestyles, dietary changes, and the tendency of women to delay childbirth have exacerbated this problem (21). Research into the etiology, inflammatory factors, immune markers, and metabolic disorders of infertility has become essential for guiding clinical treatment. In many studies related to infertility, besides the expression of genes and proteins as well as inflammatory and immune factors, nutrition is recognized as fundamental for optimal fertility and healthy offspring. Researchers have paid particular attention to dietary intake of energy, vitamins, and antioxidants. Compared with antioxidants, excessive oxidative stress (OS) can trigger various female reproductive disorders, which will lead to gynecological diseases and female infertility (22).

OS is caused by an imbalance between reactive oxygen species (ROS) and protective antioxidants, which can affect female reproductive outcomes (23). Then, OS resulted from excessive accumulation of ROS will lead to abnormal follicular atresia and meiosis, low fertilization rate, delayed embryonic development, and reproductive diseases including polycystic ovary syndrome (PCOS), ovarian endometriosis, etc. (24). Moderate intake of antioxidants might reduce the bioavailability of toxic oxidants to protect oocyte maturation (25). VC is a crucial antioxidant and free radical scavenger, soluble in water and synthesized by plants and most animals, absorbed mainly in the distal ileum. Because individuals lack L-gulono-1,4-lactone oxidase, VC require exogenous supplementation through diet or other ways (26). It aids collagen, hormone, and carnitine synthesis, and immune system function (27, 28). Its antioxidant activity depends on its capability of removing ROS, inhibiting the activity of NF- κB and reducing target gene expression (29). VC can effectively impede the peroxidation process by suppressing ROS accumulation to protect ovarian tissue from OS damage (30). Further experimental research proves that the protective mechanism of VC after exposure to oxidative damage is to diminish cell apoptosis by reducing the expression of caspase 3 or 8 and the levels of anti-Mullerian hormone in rat ovarian and uterine tissues (31). Wang et al. confirmed small-molecule compounds VC and AM580 in combination (V580) for inducing differentiation of female embryonic stem cells, which promoted meiosis progression and folliculogenesis of primordial germ cells (32). Therefore, it is indispensable to take consideration of dietary VC consumption and supplementation during the peri-pregnancy period (33).

The growing recognition of the importance of mental health in modern society is underscored by evidence consistently linking elevated stress levels to adverse reproductive health outcomes, such as anovulation, oligomenorrhea, infertility, and pregnancy complications (34–37). A meta-analysis encompassing 124,556 women revealed that those with depression face a 40% increased risk of infertility (38). In alignment with this, Maroufizadeh et al. identified prolonged infertility duration and unsuccessful treatment as significant factors associated with depressive symptoms (39). Mechanistically, neuroinflammation in the brains of depressed individuals promotes the generation of ROS, which has been closely linked to infertility (23, 40). Further investigating this association, scientists have utilized the PHQ-9 score to assess depressive symptoms in infertile patients to explore the value of dietary nutrition in mitigating these symptoms (41).

Current evidence suggests a potential pathway through which VC may influence female fertility via neuroendocrine mechanisms. Specifically, Travica et al. and Moritz et al. demonstrated that VC as an essential antioxidant and enzymatic cofactor, facilitates neurotransmitter synthesis and exerts neuroprotective effects, which may contribute to its antidepressant-like properties (42, 43). Furthermore, it reported that depression and chronic stress can lead to dysregulation of the hypothalamic-pituitary-adrenal (HPA) axis and aberrant cortisol secretion (44). Aberrant HPA axis activity directly suppresses hypothalamic-pituitary-ovarian (HPO) axis function, resulting in ovulatory disorders and poor *in vitro* fertilization (IVF) outcome (45). The clinical relevance of this pathway is supported by robust macro-level evidence. This evidence confirms that failed attempts to conceive can be attributed to adverse psychological status, which suggests the pivotal role of psycho-neuroendocrine mechanisms within the broader physiological cascade (46).

Obesity is increasing worldwide and has detrimental influences on the female reproductive endocrine system (47). Obese women were prone to experience perturbations of the HPO axis and menstrual dysfunction, such as anovulation and infertility (14). Oocytes, pre-implantation embryonic trophoblasts, and endometrium were mainly affected by obesity, which caused unsatisfied outcomes of IVF (1). Broughton et al. considered that high free fatty acids increased ROS damage to non-adipocytes, followed by mitochondrial and endoplasmic reticulum stress and cell apoptosis (48). The experimental results of Julie S Rhee and other scholars indicated that the defect of endometrial stromal cell decidualization led to impaired endometrial receptivity and poor implantation, which had a negative impact on the reproductive outcomes of obese women (49). Currently, more and more research was focused on interventions to reduce the effect of obesity on infertility, such as weight loss and physical activity (50, 51). However, it was high time that we figured out a more potent dietary nutrient to protect females against infertility because patients have limited benefits from these interventions. We took BMI as an observation indicator to explore the beneficial dietary nutrients for female infertility patients, which was particularly necessary.

Obesity, particularly visceral adiposity, is characterized by chronic low-grade inflammation, driven by proinflammatory cytokines such as TNF-α and IL-6. These mediators impair insulin signaling and disrupt ovulatory function (52). As a potent antioxidant, VC may counteract these effects by reducing oxidative stress and suppressing inflammatory responses, in order to improve insulin sensitivity and support metabolic health (53). Furthermore, VC is an essential cofactor in carnitine synthesis, which is required for mitochondrial fatty acid oxidation and energy production (54). Adequate VC levels may enhance lipid metabolism and reduce fat accumulation, as suboptimal VC status is correlated with higher body fat and waist circumference (55). Insulin resistance is a central feature of PCOS, which is a leading cause of infertility in women and is frequently associated with obesity (56). Through its antioxidant activity, VC helps protect pancreatic β-cells and insulin sensitive tissue from oxidative damage (57). Better glycemic control may reduce fat storage and attenuate obesity related endocrine disturbances that adversely affect reproductive function (58).

The advantage of this study was that it included not only the baseline characteristics of participants but also the smooth curve fitting between VC intake and female infertility, verifying the reliability of the results through sensitivity analysis. Moreover, the large sample size of the study laid a solid foundation for quantitatively evaluating the relationship between dietary VC intake and female infertility. An increase in VC intake was significantly negatively correlated with the risk of female infertility. The results of this study demonstrated that a daily VC intake of reached 132.7 mg might maximize the reduction in the likelihood of infertility in women aged from 18 to 34. Too little VC intake will increase the risk of infertility. However, VC daily doses above 400 mg have no evident value, 1 g or more intake sometimes accompanied by gastrointestinal discomfort (59–61). As a protective factor for female fertility, VC intake was inversely related to the possibility of infertility through PHQ-9 score and BMI, which indicated a potential mediating role of physical and psychological status in the relationship between VC intake and female infertility. This study aimed to investigate the potential underlying mechanisms involved. However, these mechanisms have not yet been fully elucidated; an alternative explanation is that higher vitamin C intake may simply be indicative of a generally healthier dietary pattern. Further investigation revealed that increasing VC intake had a more pronounced effect on enhancing fertility in women with lower educational attainment, possibly due to higher dietary balance requirements in those with higher education levels (62).

This study also has several limitations. Firstly, the findings are primarily generalizable to the US population and may not extend to other racial or ethnic groups. Secondly, while key confounders are adjusted for, residual confounding may persist despite DAG-guided covariate adjustment. Thirdly, dietary VC intake is assessed via a single 24-h dietary recall interview per participant, which is susceptible to recall bias and may not fully represent habitual intake. Given its cross-sectional design, this study cannot establish causality; the mediation analysis aims to explore the potential association between dietary VC intake and female infertility. Although we applied female infertility definition, the absence of questionnaire-based data may have introduced potential misclassification, which would typically attenuate true effect sizes. Future research is warranted to explore the effects of a wider range of dietary supplements and the synergistic interactions among them. Investigation into how supplement combinations collectively influence reproductive outcomes holds considerable promise for the development of more effective nutritional interventions.

## Conclusions

This study figured out that dietary VC intake might be significantly beneficial to decrease infertility rate through two potential mechanisms (depression degree and BMI) in females aged between 18 and 34. However, it is still indispensable for us to conduct further RCTs to confirm whether a higher dietary VC intake could prevent those women females aged between 18 and 34 from infertility. In addition, future clinical studies should concentrate more on the relationship among dietary, mental health, physical status and individual characteristics of women, which may protect them against infertility.

## Data Availability

The original contributions presented in the study are included in the article/supplementary material, further inquiries can be directed to the corresponding author.

## References

[1] Broughton DE Moley KH. Obesity and female infertility: potential mediators of obesity's impact. Fertil Steril. (2017) 107:840–7. 10.1016/j.fertnstert.2017.01.017 28292619

[2] Caballero B. Humans against obesity: who will win? Adv Nutr. (2019) 10:S4–s9. 10.1093/advances/nmy055 30721956 PMC6363526

[3] Carson SA Kallen AN. Diagnosis and management of infertility: a review. JAMA. (2021) 326:65–76. 10.1001/jama.2021.4788 34228062 PMC9302705

[4] Bala R Singh V Rajender S Singh K. Environment, lifestyle, and female infertility. Reprod Sci. (2021) 28:617–38. 10.1007/s43032-020-00279-3 32748224

[5] González-Rodríguez LG López-Sobaler AM Perea Sánchez JM Ortega RM. [Nutrition and fertility]. Nutr Hosp. (2018) 35:7–10. 10.20960/nh.2279 30351153

[6] Humeniuk E Pucek W Wdowiak A Filip M Bojar I Wdowiak A. Supporting the treatment of infertility using psychological methods. Ann Agric Environ Med. (2023) 30:581–6. 10.26444/aaem/171874 38153057

[7] Matzuk MM Lamb DJ. The biology of infertility: research advances and clinical challenges. Nat Med. (2008) 14:1197–213. 10.1038/nm.f.1895 18989307 PMC3786590

[8] Passet-Wittig J Greil AL. Factors associated with medical help-seeking for infertility in developed countries: a narrative review of recent literature. Soc Sci Med. (2021) 277:113782. 10.1016/j.socscimed.2021.113782 33895708

[9] Panchenko PE Lemaire M Fneich S Voisin S Jouin M Junien C. [Epigenetics and Nutrition: maternal nutrition impacts on placental development and health of offspring]. Biol Aujourdhui. (2015) 209:175–87. 10.1051/jbio/2015021 26514387

[10] Ciebiera M Esfandyari S Siblini H Prince L Elkafas H Wojtyła C. Nutrition in gynecological diseases: current perspectives. Nutrients. (2021) 13:1178. 10.3390/nu13041178 33918317 PMC8065992

[11] Showell MG Mackenzie-Proctor R Jordan V Hart RJ. Antioxidants for female subfertility. Cochrane Database Syst Rev. (2017) 7:Cd007807. 10.1002/14651858.CD007807.pub3 28752910 PMC6483341

[12] DiTroia SP Percharde M Guerquin MJ Wall E Collignon E Ebata KT. Maternal vitamin C regulates reprogramming of DNA methylation and germline development. Nature. (2019) 573:271–5. 10.1038/s41586-019-1536-1 31485074 PMC8423347

[13] Ruder EH Hartman TJ Reindollar RH Goldman MB. Female dietary antioxidant intake and time to pregnancy among couples treated for unexplained infertility. Fertil Steril. (2014) 101:759–66. 10.1016/j.fertnstert.2013.11.008 24355050 PMC3943921

[14] Silvestris E de Pergola G Rosania R Loverro G. Obesity as disruptor of the female fertility. Reprod Biol Endocrinol. (2018) 16:22. 10.1186/s12958-018-0336-z 29523133 PMC5845358

[15] Yu K Liu X Alhamzawi R Becker F Lord J. Statistical methods for body mass index: a selective review. Stat Methods Med Res. (2018) 27:798–811. 10.1177/0962280216643117 27072505

[16] Carr AC Block G Lykkesfeldt J. Estimation of vitamin C intake requirements based on body weight: implications for obesity. Nutrients. (2022) 14:1460. 10.3390/nu14071460 35406073 PMC9003354

[17] Kundakovic M Rocks D. Sex hormone fluctuation and increased female risk for depression and anxiety disorders: from clinical evidence to molecular mechanisms. Front Neuroendocrinol. (2022) 66:101010. 10.1016/j.yfrne.2022.101010 35716803 PMC9715398

[18] Kroenke K Spitzer RL Williams JB. The PHQ-9: validity of a brief depression severity measure. J Gen Intern Med. (2001) 16:606–13. 10.1046/j.1525-1497.2001.016009606.x 11556941 PMC1495268

[19] Zhou Y Sun Z Song J. Research progress on the impact of anxiety and depression on embryo transfer outcomes of *in vitro* fertilization. Zhejiang Da Xue Xue Bao Yi Xue Ban. (2023) 52:61–7. 10.3724/zdxbyxb-2022-0473 37283119 PMC10293778

[20] Han QQ Wu PF Li YH Cao Y Chen JG Wang F. SVCT2-mediated ascorbic acid uptake buffers stress responses via DNA hydroxymethylation reprogramming of S100 calcium-binding protein A4 gene. Redox Biol. (2022) 58:102543. 10.1016/j.redox.2022.102543 36436457 PMC9694147

[21] Vander Borght M Wyns C. Fertility and infertility: definition and epidemiology. Clin Biochem. (2018) 62:2–10. 10.1016/j.clinbiochem.2018.03.012 29555319

[22] Vašková J Klepcová Z Špaková I Urdzík P Štofilová J Bertková I. The importance of natural antioxidants in female reproduction. Antioxidants. (2023) 12:907. 10.3390/antiox12040907 37107282 PMC10135990

[23] Adeoye O Olawumi J Opeyemi A Christiania O. Review on the role of glutathione on oxidative stress and infertility. JBRA Assist Reprod. (2018) 22:61–6. 10.5935/1518-0557.20180003 29266896 PMC5844662

[24] Wang L Tang J Wang L Tan F Song H Zhou J. Oxidative stress in oocyte aging and female reproduction. J Cell Physiol. (2021) 236:7966–83. 10.1002/jcp.30468 34121193

[25] Silvestris E Lovero D Palmirotta R. Nutrition and female fertility: an interdependent correlation. Front Endocrinol (Lausanne). (2019) 10:346. 10.3389/fendo.2019.00346 31231310 PMC6568019

[26] Li XY Meng L Shen L Ji HF. Regulation of gut microbiota by vitamin C, vitamin E and β-carotene. Food Res Int. (2023) 169:112749. 10.1016/j.foodres.2023.112749 37254375

[27] Doseděl M Jirkovský E Macáková K Krčmová LK Javorská L Pourová J. Vitamin C-sources, physiological role, kinetics, deficiency, use, toxicity, and determination. Nutrients. (2021) 13:615. 10.3390/nu13020615 33668681 PMC7918462

[28] Milani GP Macchi M Guz-Mark A. Vitamin C in the treatment of COVID-19. Nutrients. (2021) 13:1172. 10.3390/nu13041172 33916257 PMC8065688

[29] Pérez-Torres I Castrejón-Téllez V Soto ME Rubio-Ruiz ME Manzano-Pech L Guarner-Lans V. Oxidative stress, plant natural antioxidants, and obesity. Int J Mol Sci. (2021) 22:1786. 10.3390/ijms22041786 33670130 PMC7916866

[30] Agarwal A Gupta S Sharma RK. Role of oxidative stress in female reproduction. Reprod Biol Endocrinol. (2005) 3:28. 10.1186/1477-7827-3-28 16018814 PMC1215514

[31] Saygin M Ozmen O Erol O Ellidag HY Ilhan I. Aslankoc R. The impact of electromagnetic radiation (245 GHz, Wi-Fi) on the female reproductive system: the role of vitamin C. Toxicol Ind Health. (2018) 34:620–30. 10.1177/0748233718775540 29848237

[32] Wang H Liu L Liu C Wang L Chen J Wang H. Induction of meiosis by embryonic gonadal somatic cells differentiated from pluripotent stem cells. Stem Cell Res Ther. (2021) 12:607. 10.1186/s13287-021-02672-4 34930450 PMC8686525

[33] Camarena V Wang G. The epigenetic role of vitamin C in health and disease. Cell Mol Life Sci. (2016) 73:1645–58. 10.1007/s00018-016-2145-x 26846695 PMC4805483

[34] Jain P Chauhan AK Singh K Garg R Jain N Singh R. Correlation of perceived stress with monthly cyclical changes in the female body. J Family Med Prim Care. (2023) 12:2927–33. 10.4103/jfmpc.jfmpc_874_23 38186841 PMC10771141

[35] Meczekalski B Niwczyk O Bala G Szeliga A. Stress, kisspeptin, and functional hypothalamic amenorrhea. Curr Opin Pharmacol. (2022) 67:102288. 10.1016/j.coph.2022.102288 36103784

[36] Schliep KC Hinkle SN Kim K Sjaarda LA Silver RM Stanford JB. Prospectively assessed perceived stress associated with early pregnancy losses among women with history of pregnancy loss. Hum Reprod. (2022) 37:2264–74. 10.1093/humrep/deac172 35972454 PMC9802052

[37] Vigil P Meléndez J Soto H Petkovic G Bernal YA Molina S. Chronic stress and ovulatory dysfunction: implications in times of COVID-19. Front Glob Womens Health. (2022) 3:866104. 10.3389/fgwh.2022.866104 35677754 PMC9168655

[38] Nik Hazlina NH Norhayati MN Shaiful Bahari I Nik Muhammad Arif NA. Worldwide prevalence, risk factors and psychological impact of infertility among women: a systematic review and meta-analysis. BMJ Open. (2022) 12:e057132. 10.1136/bmjopen-2021-057132 35354629 PMC8968640

[39] Maroufizadeh S Omani-Samani R Almasi-Hashiani A Amini P Sepidarkish M. The reliability and validity of the Patient Health Questionnaire-9 (PHQ-9) and PHQ-2 in patients with infertility. Reprod Health. (2019) 16:137. 10.1186/s12978-019-0802-x 31500644 PMC6734346

[40] Riveros ME Ávila A Schruers K Ezquer F. Antioxidant biomolecules and their potential for the treatment of difficult-to-treat depression and conventional treatment-resistant depression. Antioxidants. (2022) 11:540. 10.3390/antiox11030540 35326190 PMC8944633

[41] Kris-Etherton PM Petersen KS Hibbeln JR Hurley D Kolick V Peoples S. Nutrition and behavioral health disorders: depression and anxiety. Nutr Rev. (2021) 79:247–60. 10.1093/nutrit/nuaa025 32447382 PMC8453603

[42] Moritz B Schmitz AE Rodrigues ALS Dafre AL Cunha MP. The role of vitamin C in stress-related disorders. J Nutr Biochem. (2020) 85:108459. 10.1016/j.jnutbio.2020.108459 32745879

[43] Travica N Ried K Sali A Scholey A Hudson I Pipingas A. Vitamin C status and cognitive function: a systematic review. Nutrients. (2017) 9:960. 10.3390/nu9090960 28867798 PMC5622720

[44] Hannibal KE Bishop MD. Chronic stress, cortisol dysfunction, and pain: a psychoneuroendocrine rationale for stress management in pain rehabilitation. Phys Ther. (2014) 94:1816–25. 10.2522/ptj.20130597 25035267 PMC4263906

[45] Pandey AK Gupta A Tiwari M Prasad S Pandey AN Yadav PK. Impact of stress on female reproductive health disorders: possible beneficial effects of shatavari (*Asparagus racemosus*). Biomed Pharmacother. (2018) 103:46–9. 10.1016/j.biopha.2018.04.003 29635127

[46] Dube L Bright K Hayden KA Gordon JL. Efficacy of psychological interventions for mental health and pregnancy rates among individuals with infertility: a systematic review and meta-analysis. Hum Reprod Update. (2023) 29:71–94. 10.1093/humupd/dmac034 36191078

[47] Talmor A Dunphy B. Female obesity and infertility. Best Pract Res Clin Obstet Gynaecol. (2015) 29:498–506. 10.1016/j.bpobgyn.2014.10.014 25619586

[48] Broughton DE Jungheim ES A. Focused look at obesity and the preimplantation trophoblast. Semin Reprod Med. (2016) 34:5–10. 10.1055/s-0035-1570032 26696274

[49] Rhee JS Saben JL Mayer AL Schulte MB Asghar Z Stephens C. Diet-induced obesity impairs endometrial stromal cell decidualization: a potential role for impaired autophagy. Hum Reprod. (2016) 31:1315–26. 10.1093/humrep/dew048 27052498 PMC4871191

[50] Gambineri A Laudisio D Marocco C Radellini S Colao A Savastano S. Female infertility: which role for obesity? Int J Obes Suppl. (2019) 9:65–72. 10.1038/s41367-019-0009-1 31391925 PMC6683114

[51] Hakimi O Cameron LC. Effect of exercise on ovulation: a systematic review. Sports Med. (2017) 47:1555–67. 10.1007/s40279-016-0669-8 28035585

[52] Ellulu MS Patimah I. Khaza'ai H, Rahmat A, Abed Y. Obesity and inflammation: the linking mechanism and the complications. Arch Med Sci. (2017) 13:851–63. 10.5114/aoms.2016.58928 28721154 PMC5507106

[53] Ellulu MS Rahmat A Patimah I. Khaza'ai H, Abed Y. Effect of vitamin C on inflammation and metabolic markers in hypertensive and/or diabetic obese adults: a randomized controlled trial. Drug Des Devel Ther. (2015) 9:3405–12. 10.2147/DDDT.S83144 26170625 PMC4492638

[54] Rebouche CJ. Ascorbic acid and carnitine biosynthesis. Am J Clin Nutr. (1991) 54:1147s−52. 10.1093/ajcn/54.6.1147s 1962562

[55] Johnston CS Corte C Swan PD. Marginal vitamin C status is associated with reduced fat oxidation during submaximal exercise in young adults. Nutr Metab. (2006) 3:35. 10.1186/1743-7075-3-35 16945143 PMC1564400

[56] Zeng X Xie YJ Liu YT Long SL Mo ZC. Polycystic ovarian syndrome: correlation between hyperandrogenism, insulin resistance and obesity. Clin Chim Acta. (2020) 502:214–21. 10.1016/j.cca.2019.11.003 31733195

[57] Mason SA Rasmussen B van Loon LJC Salmon J Wadley GD. Ascorbic acid supplementation improves postprandial glycaemic control and blood pressure in individuals with type 2 diabetes: findings of a randomized cross-over trial. Diabetes Obes Metab. (2019) 21:674–82. 10.1111/dom.13571 30394006

[58] Vatier C Christin-Maitre S Vigouroux C. Role of insulin resistance on fertility - focus on polycystic ovary syndrome. Ann Endocrinol. (2022) 83:199–202. 10.1016/j.ando.2022.04.004 35436501

[59] Bsoul SA Terezhalmy GT. Vitamin C in health and disease. J Contemp Dent Pract. (2004) 5:1–13. 10.5005/jcdp-5-2-1 15150630

[60] Levine M Conry-Cantilena C Wang Y Welch RW Washko PW Dhariwal KR. Vitamin C pharmacokinetics in healthy volunteers: evidence for a recommended dietary allowance. Proc Natl Acad Sci USA. (1996) 93:3704–9. 10.1073/pnas.93.8.3704 8623000 PMC39676

[61] Levine M Rumsey SC Daruwala R Park JB Wang Y. Criteria and recommendations for vitamin C intake. Jama. (1999) 281:1415–23. 10.1001/jama.281.15.1415 10217058

[62] Rippin HL Hutchinson J Greenwood DC Jewell J Breda JJ Martin A. Inequalities in education and national income are associated with poorer diet: pooled analysis of individual participant data across 12 European countries. PLoS ONE. (2020) 15:e0232447. 10.1371/journal.pone.0232447 32379781 PMC7205203

