# Supplementary figures and images for: Depression score and body mass index mediate the association between dietary vitamin C intake and female infertility: a study based on NHANES 2013–2018
Source: Front Nutr. 2025 Sep 10;12:1650311. doi: 10.3389/fnut.2025.1650311 (PMC12457304; doi:10.3389/fnut.2025.1650311)

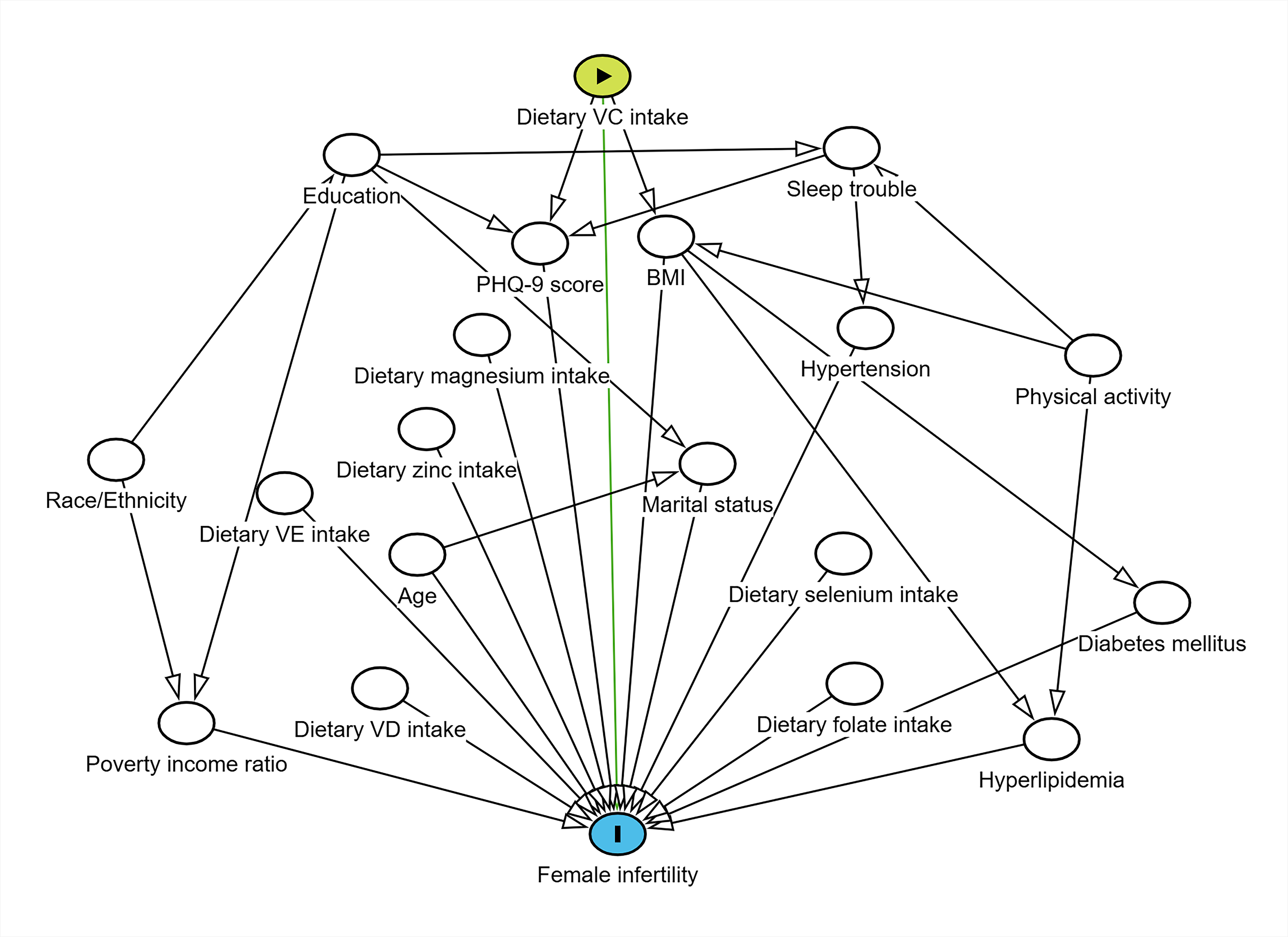

Supplement: Supplementary file 1 [file Image_1.tif]
